# Association of County Race and Ethnicity Characteristics With Number of Insurance Carriers and Insurance Network Breadth

**DOI:** 10.1001/jamanetworkopen.2022.7404

**Published:** 2022-04-27

**Authors:** Sebastian Linde, Leonard E. Egede

**Affiliations:** 1 Medical College of Wisconsin, Division of General Internal Medicine, Department of Medicine, Milwaukee; 2 Center for Advancing Population Sciences, Medical College of Wisconsin, Milwaukee

## Abstract

**Importance:**

While the Affordable Care Act (ACA) set out to eliminate insurer discrimination based on preexisting conditions, the ACA health exchanges allow insurers to select what markets to enter and afford them great freedom on how they design their physician networks. Strategic market participation and physician network design based on population race, ethnicity, and health characteristics may give rise to a present-day form of redlining within health insurance markets—ie, a systematic underprovision of insurance plans and in-network practitioners within areas that are populated with higher proportions of non-Hispanic Black residents.

**Objective:**

To examine if markets with relatively higher non-Hispanic Black populations have systematically fewer insurers and lower network inclusion of physicians residing within these areas.

**Design, Setting, and Participants:**

This cohort study conducted a regression analysis of the US ACA health insurance exchange marketplace across 34 states with federal exchanges and physicians located within the 500 most populous US cities in 2014. County-level data were sourced from individual market and issuer enrollment databases and county health rankings; census tract data came from a national database of physician networks in 2014 marketplace plans, US Census Bureau data, and the Centers for Disease Control and Prevention’s PLACES database. Adjustment was made for a rich set of county (or census tract) controls and state fixed effects to capture broad market and/or policy differences across states. Analyses were performed in June 2021.

**Main Outcomes and Measures:**

The raw count of insurers within a county and the mean percentage of insurance networks that physicians participate in within each census tract.

**Results:**

A total of 2270 counties were examined within our first analyses. In the counties analyzed, a mean (SD) of 23.0% (3.2%) of the population was aged 18 years or younger, and a mean (SD) of 11.0% (15.8%) of the population had non-Hispanic Black race and ethnicity. For the second analysis, 16 006 to 25 096 census tracts were examined (depending on physician specialty). With adjustment for population size, age, and race and ethnicity, a 1-SD increase in the county non-Hispanic Black population was associated with a 14.1% reduction in the number of insurers (mean [SE] marginal effect size, −2.18 [0.13]; *P* < .001). Accounting for additional county-level risk selection controls and state fixed effects, a 1-SD increase in the non-Hispanic Black population was associated with a 2.3% reduction in available insurers (marginal effect size, −0.36 [0.17]; *P* = .04). For practitioners network breadth inclusion, a 1-SD increase in the non-Hispanic Black population was associated with a 15.8% (marginal effect size, −0.32 [0.01]; *P* < .001) to 24.7% (marginal effect size, −0.14 [0.02]; *P* < .001) reduction in the physicians’ network participation depending on their specialty. Adjusting for additional state fixed effects yielded estimates of 6% (marginal effect size, −0.08 [0.01]; *P* < .001) to 13.5% (marginal effect size, −0.12 [0.02]; *P* < .001) reductions in practitioner network participation.

**Conclusions and Relevance:**

These findings suggest that strategic decisions by insurers may contribute toward markets with higher racial or ethnic minority populations having systematically fewer participating insurers, as well as a higher prevalence of local physicians not included in coverage networks. These findings call for further examination of potential insurance redlining within the ACA marketplaces.

## Key Points

**Question:**

Are strategic decisions made by insurers in the Affordable Care Act health exchanges about what markets to enter and what physicians to include within their networks associated with the underprovision of insurance plans and in-network options in areas with higher non-Hispanic Black population shares?

**Findings:**

In this cohort study of health insurance exchange data with county and census tract controls, a 1-SD increase in county non-Hispanic Black population was associated with a 14.1% reduction in the number of insurers offering plans, and a 1-SD increase in the census-tract non-Hispanic Black population was associated with a 15.8% to 24.7% reduction in physicians’ insurance network participation.

**Meaning:**

These findings suggest that strategic decisions by insurers in the Affordable Care Act marketplaces may contribute to fewer insurance options being available in markets with higher racial or ethnic minority populations.

## Teaser

This cohort study of health insurance exchange marketplace data examines the association of racial and ethnic makeup of geographical areas with number of insurers and network inclusion of physicians within these areas following implementation of the US Affordable Care Act.

## Introduction

Structural racism, the ways in which societies foster discrimination through mutually reinforcing inequitable systems, has emerged as an important social risk factor and contributor to poor health outcomes for populations historically marginalized on the basis of race or ethnicity.^1,2,3,4^ An important component of structural racism that has been inadequately studied is redlining. Redlining has referred to the practice of systematically denying credit access and insurance for borrowers in neighborhoods that were economically disadvantaged and that were inhabited primarily by Black Americans.^5,6^ In a study of historically redlined areas across 115 cities, Mitchell and Franco^7^ showed that many of these areas are to this day disproportionately economically disadvantaged, while Richardson and colleagues^5^ further showed that these structural disparities appear to adversely affect the health of residents within these areas. Other recent work also showed that individuals living in historically disadvantaged areas continue to experience residual effects from discriminatory practices, such as redlining, on their health to this day.^8,9,10,11^

An important link between historical redlining and health outcomes is likely related to health insurers’ strategic considerations about what markets to participate within, how to design coverage plans, and what physicians to include within their networks based on neighborhood characteristics. To the extent that these decisions are associated with the racial or ethnic makeup of these markets, we refer to this as insurance redlining (where *redlining* is used metaphorically to describe associations of coverage decisions with neighborhood demographics rather than disclosed evidence of explicit racial or ethnic discrimination). While prior work has helped identify race-based access gaps when it comes to insurance coverage,^12,13^ primary care access,^14,15^ and even the strategic location choice of where to set up freestanding emergency departments,^16^ much less is known about the extent to which the practice of insurance redlining may be manifesting itself on the Affordable Care Act (ACA) health exchanges. Thus, it is critical to examine whether current strategic considerations made by health insurers about what markets to enter and how to design plans perpetuate existing structural inequities experienced by racial and ethnic minority populations. In order to address this gap in knowledge, we studied insurer market participation and health care network design within the ACA market exchanges in 2014. In particular, we set out to examine 2 hypotheses: (1) there exists a significant negative association between county-level proportion of non-Hispanic Black individuals and insurer market participation and (2) there exists a significant negative association between the proportion of non-Hispanic Black individuals and the inclusion of local physicians in insurance networks at the census tract level.

The first hypothesis is important to examine because limited competition within the ACA individual insurance markets can result in patients having to face higher costs of coverage. This inverse association between insurance premiums and competition has been noted by a number of studies.^17,18^ The second hypothesis is similarly important to examine because low inclusion of health care professionals within insurance networks may limit the services that are proximate to and included within the insurance plans of local beneficiaries.

## Methods

### Study Sample

In this cohort study of the post-ACA US health insurance marketplace, we combined data from a number of sources in order to construct 2 analysis samples. The first of these drew from the Health Insurance Exchange Compare Public Use Files from the HIX Compare Individual Market database for 2014.^19^ These files allowed us to identify insurers’ county-level market participation decisions across 34 US states within the individual ACA marketplace. These data were combined with county-level demographic data from the County Health Rankings Project.^20^ Our final study sample consisted of 2270 counties (see eFigure 1 and eTable 1 in the Supplement).

The second set of data included census tract data drawn from the US Census Bureau, which we sourced from the Opportunity Insight Project, and public health data from the US Centers for Disease Control and Prevention’s PLACES database, which covers 500 of the largest cities in the US nationwide.^21,22^ These data were linked with plan-specific physician network data from the Leonard Davis Institute national database of physician networks in 2014 marketplace silver plans. These data allowed us to identify health care professionals by specialty and location of their practice. In our analysis we have concentrated on the specialties of family medicine, general practice, internal medicine, and pediatrics. Because of variation in the amount of specialists in different fields across census tracts, we used separate samples for each specialty. This resulted in 4 samples for our analysis: 25 096 census tracts for family medicine, 16 006 census tracts for general practice, 24 613 tracts for internal medicine, and 23 410 for pediatricians (eFigure 2 and eTable 2 in the Supplement).

We used 2 different samples because, first, our market participation analyses sought to capture insurers’ participation decisions at the level of their participation or entry decision, which required looking at the county-level data. For the second set of analyses, we were interested in examining the breadth of insurance plan inclusion of health care professionals residing within specific communities or neighborhoods, which required a sample constructed at the more granular census tract level.

This study was based on public and deidentified data and did not constitute human subjects research as defined by 45 CFR §46.102. We followed the Strengthening the Reporting of Observational Studies in Epidemiology (STROBE) reporting guideline for cohort studies.

### Study Variables

#### Outcome Measures

For our first set of analyses, we used the number of insurance carriers within a given county as our main outcome measure. This was simply a raw count of insurers within a county. For our second set of analyses, we computed the number of unique plans on offer within each county, and then computed the percentage of those networks that each health care professional was a part of: insurance network breadth = (total number of networks clinician is a part of)/(total number of networks available in the county)*.* The mean averages of these percentages were then calculated on the census tract level for each specialty field (family medicine, general practice, and internal medicine) in order to obtain our census tract mean practitioner network breadth inclusion measures.

#### Independent Variable

Our main variable of interest was the percentage of non-Hispanic Black individuals residing within a given geographical area. For our first set of analyses (our insurer market prevalence analysis), the geographic unit was at the county level; in our second set of analyses (insurance network inclusion analysis) the measure is at the more granular census tract level.

### Other Covariates

We utilized a rich set of additional control variables for both our county-level and census tract–level analyses. At the county level, these consisted of: (1) US Census Bureau population estimates for the percentage of residents ages 65 years and older, 18 years and younger, percentage Hispanic and Asian residents, and overall county population; (2) National Center for Health Statistics data on premature death, defined as the years of potential life lost before age 75 years per 100 000 population; (3) diabetes prevalence; (4) Dartmouth Atlas Data on preventable hospital stays, defined as the number of hospital stays for ambulatory care–sensitive conditions per 1000 Medicare enrollees; and (5) US Census Bureau’s Small Area Income and Poverty Estimates program data on median household income. These were all sourced from the 2014 County Health Rankings database.^20^

At the census tract level, our additional controls consisted of (1) percentage of Hispanic and Asian residents, population density, population count, and median household income, which are all sourced from Opportunity Insights and based on the 2010 US Census; and (2) diabetes prevalence, asthma prevalence, coronary heart disease prevalence, and annual checkup prevalence, which we sourced from the Centers for Disease Control and Prevention 2014 PLACES database.^22^

### Statistical Analysis

We used multivariable regression methods to perform 2 sets of analyses. The first estimated the association of the county-level prevalence of non-Hispanic Black residents with the number of insurance carriers that enter a given market; the second estimated the association of the census tract–level prevalence of non-Hispanic Black residents with insurance network breadth. Within both sets of analyses, we made successive adjustments for additional controls pertaining to area demographics, health, and median household income, as well as state indicators. These additional controls were included to adjust for other factors that may affect risk selection by insurers in terms of their market participation and network design, while the state indicators (or fixed effects) were included in order to capture latent state-level policies or characteristics that might otherwise confound our analyses. (Additional regression model specification details are provided in eMethods, and additional sensitivity analyses are provided in eTables 3 through 6 in the Supplement.) All analyses were performed using Stata MP version 17 (StataCorp). Statistical significance was assigned at the level of 95% CIs (*P* < .05), and hypothesis tests were 2-sided. We also calculated the percentage reduction of insurers given a 1-SD increase in the county non-Hispanic Black population after adjusting for population size, age, and race and ethnicity using the formula: (marginal effect size estimate × SD)/mean.

## Results

### Sample Characteristics

In the first sample (ie, the county-level insurer prevalence analysis), the mean (SD) number of insurance carriers within a county was 2.5 (1.2) and Asian residents constituted a mean (SD) of 1.0% (1.6%) of the population; non-Hispanic Black residents, 11.0% (15.8%); and Hispanic residents, 8.1% (13.2%) (Table 1). The county health measures indicated a mean average of 8311.4 (2226.2) premature–death years, a diabetes prevalence of 11.1% (2.1%), and 78.7 (27.3) preventable hospital stays per 1000 Medicare enrollees.

**Table 1.** Summary Statistics Across County and Census Tract Samples

Characteristics	No.	Residents, Mean (SD), %
**County-level insurer participation analysis**		
No. of health insurance carriers, mean (SD)	2270	2.5 (1.2)
Population count, No.	2270	89 687.1 (244 600.3)
Age		
≤18 y	2270	23.0 (3.2)
≥65 y	2270	16.6 (4.2)
Race or ethnicity		
Asian	2270	1.0 (1.6)
Hispanic	2270	8.1 (13.2)
Non-Hispanic Black	2270	11.0 (15.8)
Non-Hispanic White	2270	77 (20)
Rural	2270	57.3 (30.3)
Premature death, No.	2270	8311.4 (2226.2)
Diabetes prevalence	2270	11.1 (2.1)
Preventable hospital stays, No.	2270	78.7 (27.3)
Median household income, $	2270	43 613.9 (10 899.1)
**Census tract network breadth analysis**		
Network breadth		
Family medicine	25 096	34.8 (20.9)
General practice	16 006	24.2 (22.0)
Internist	24 613	35.7 (20.0)
Pediatrician	23 410	37.7 (21.1)
Race and ethnicity		
Asian	26 432	6.0 (10.1)
Hispanic	26 432	23.5 (24.6)
Non-Hispanic Black	26 432	20.4 (26.5)
Population, No.		
Count	26 432	3830.6 (1929.2)
Density	26 432	10 665.5 (17 590.8)
Diabetes	26 432	10.7 (4.1)
Asthma	26 432	9.7 (2.0)
Coronary heart disease	26 432	5.8 (2.0)
Annual checkup	26 432	68.2 (6.4)
Median household income, $	26 432	57 204.8 (29 961.8)

For the census tract insurer network breadth analysis sample, the mean (SD) network breadth was 34.8% (20.9%) for physicians with a specialty of family medicine, 24.2% (22.0%) for physicians with a general practice specialty, 35.7% (20.0%) for internists, and 37.7% (21.1%) for pediatricians. Census tracts had a mean (SD) of 6.0% (10.0%) Asian residents, 20.4% (26.5%) non-Hispanic Black residents, and 23.5% (24.6%) Hispanic residents. The diabetes prevalence was 10.7% (4.1%), asthma prevalence was 9.7% (2.0%), coronary heart disease prevalence was 5.8% (2.0), and annual checkup prevalence was 68.2% (6.4) (eFigures 1 and 2 and eTable 2 in the Supplement).

### Market Participation of Insurance Carriers and County Race and Ethnicity

County non-Hispanic Black population was associated with fewer health insurers participating in that county (Figure). Approximately 82% of counties within our sample had between 1 and 3 carriers, indicating high market concentration.

**Figure.**
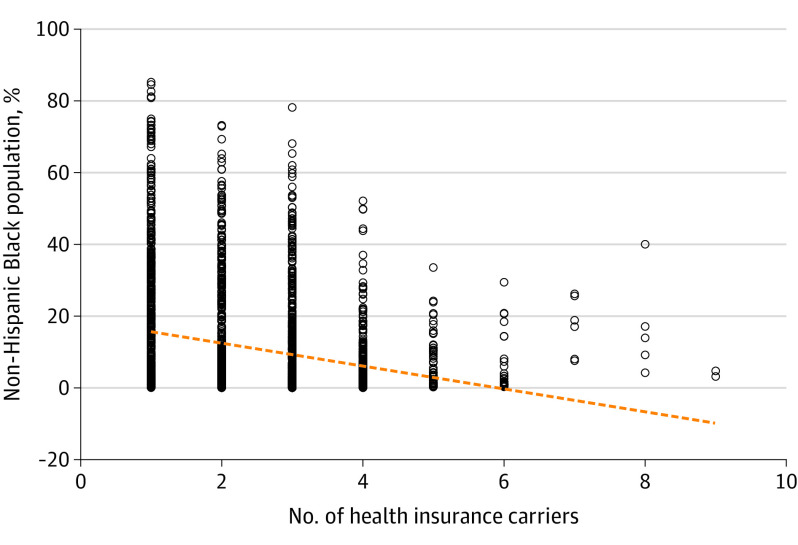
Total Health Insurance Carriers and the Percentage of Non-Hispanic Black Residents at the County Level Black circles indicate individual health insurers; dashed orange line, fitted trend.

The inverse association of non-Hispanic Black population and number of insurers persisted even after adjusting for other county characteristics that may factor into insurers’ market participation decision (Table 2). After adjusting for population size, age, and race and ethnicity, a 1-SD increase in the county non-Hispanic Black population was associated with a 14.1% reduction of insurers (marginal effect size, −2.18 [0.13]; *P* < .001). This percentage reduction was computed using the marginal effect size estimates from Table 2, together with mean and SD measures from Table 1, using the formula: (marginal effect size estimate × SD)/mean = (–2.18 × 0.16)/2.48 = –0.141. (Note that 0.16 within this example corresponds to the decimal representation of the SD of non-Hispanic Black race in Table 1.) When controlling for county median household income, public health, and care coordination factors, the prevalence of non-Hispanic Black residents remained a significant factor in insurer market participation, including after adjusting for potential risk selection by insurers. With that said, the association decreased by 4.5% (marginal effect size, –0.70 [0.18]; *P* < .001) given a 1-SD increase in the non-Hispanic Black population prevalence. Additional adjustments for state indicators along with the other controls indicated that a 1-SD increase in non-Hispanic Black population prevalence was associated with a 2.3% reduction (marginal effect size, –0.36 [0.17]; *P* = .04) in the number of insurance carriers.

**Table 2.** Regression Model Estimates from County-Level Sample Examining Association Between Total Market Entrants and the Non-Hispanic Black Population

Characteristic	No. of health insurance carriers					
	1, marginal effect size (SE)	2, marginal effect size (SE)	3, marginal effect size (SE)	4, marginal effect size (SE)	5, marginal effect size (SE)	6, marginal effect size (SE)
Non-Hispanic Black, %	–2.18 (0.13)^a^	–0.79 (0.18)^a^	–0.70 (0.18)^a^	–0.61 (0.15)^a^	–0.52 (0.17)^a^	–0.36 (0.17)^b^
Population and demographics controls						
Population count per 10 000 residents, No.	0.02 (0.00)^a^	0.01 (0.00)^a^	0.01 (0.00)^a^	0.01 (0.00)^a^	0.01 (0.00)^a^	0.01 (0.00)^a^
Age, y						
≤18	2.24 (0.94)^b^	4.81 (0.89)^a^	4.12 (0.90)^a^	0.59 (0.72)	1.54 (0.76)^b^	0.27 (0.78)
≥65	–0.76 (0.80)	3.78 (0.87)^a^	3.89 (0.88)^a^	–1.39 (0.63)^b^	–0.18 (0.68)	–0.04 (0.69)
Race and ethnicity						
Asian	4.74 (2.20)^b^	–4.88 (1.72)^a^	–6.93 (1.84)^a^	5.54 (2.14)^a^	1.32 (1.86)	–0.64 (1.88)
Hispanic	–0.37 (0.23)	–0.92 (0.22)^a^	–0.73 (0.22)^a^	–0.60 (0.22)^a^	–0.82 (0.22)^a^	–0.45 (0.23)^c^
Rural	NA	–0.63 (0.11)^a^	–0.61 (0.11)^a^	NA	–0.33 (0.08)^a^	–0.31 (0.08)^a^
Population health controls						
Premature death per 10 000 persons, No.	NA	–0.41 (0.13)^a^	–0.23 (0.13)^c^	NA	–0.39 (0.10)^a^	–0.16 (0.10)^c^
Diabetes prevalence	NA	–13.12 (1.64)^a^	–12.52 (1.64)^a^	NA	0.39 (1.56)	1.33 (1.56)
Preventable hospital stays per 100 stays, No.	NA	–0.36 (0.09)^a^	–0.32 (0.09)^a^	NA	–0.40 (0.07)^a^	–0.32 (0.07)^a^
Economic control						
Median household income per $10 000, $	NA	NA	0.10 (0.03)^a^	NA	NA	0.13 (0.03)^a^
Observations, No.	2270	2270	2270	2270	2270	2270
*R^2^*	0.19	0.28	0.28	0.59	0.60	0.61
State indicators^d^	No	No	No	Yes	Yes	Yes

Abbreviation: NA, not applicable.

^a^ *P* < .01.

^b^ *P* < .05.

^c^ *P* < .10.

^d^ Controls included population count, age 18 years and younger, age 65 years and older, Hispanic and Asian ethnicity, rural, premature death, diabetes prevalence, preventable hospital stays, and median household income. Controls include state indicators, which are binary indicators—1 for each state.

### Insurer Network Breadth and Area by Race and Ethnicity

A 1-SD increase in the non-Hispanic Black population prevalence was associated with a 15.8% (marginal effect size, –0.32 [0.01]; *P* < .001) to 24.7% (marginal effect size, –0.14 [0.02]; *P* < .001) reduction in the insurers’ network participation depending on their specialty (Table 3). In particular, we note a 24.7% network breadth decrease for family medicine specialties, a 15.8% decrease for general practices specialties, a 21.0% decrease for internists, and a 17.8% decrease for pediatricians. These results were all adjusted for population, income, and health characteristics. When making additional adjustments for state fixed effects, specialty specific reduction estimates ranged between 6% to 13.5% decreases in physician network participation given a 1-SD increase in the census tract non-Hispanic Black population prevalence.

**Table 3.** Regression Model Estimates From Census Tract Sample

Characteristic	Network breadth, marginal effect size (SE)							
	Family medicine		General practice		Internist		Pediatrician	
	1	2	3	4	5	6	7	8
Non-Hispanic Black, %	–0.32^a^ (0.01)	–0.10^a^ (0.01)	–0.14^a^ (0.02)	–0.12^a^ (0.02)	–0.28^a^ (0.01)	–0.08^a^ (0.01)	–0.25^a^ (0.01)	–0.09^a^ (0.01)
**Population and demographics controls**								
Population								
Total per 10 000	–0.01^b^ (0.01)	0.01^a^ (0.01)	0.04^a^ (0.01)	0.04^a^ (0.01)	0 (0.01)	0.02^a^ (0.01)	0 (0.01)	0.01 (0.01)
Density, No. per 10 000	0^a^ (0)	–0.01^a^ (0)	–0.01^a^ (0)	0^a^ (0)	0^a^ (0)	–0.01^a^ (0)	–0.01^a^ (0)	–0.01^a^ (0)
Race and/or ethnicity								
Asian	–0.50^a^ (0.02)	–0.14^a^ (0.02)	–0.27^a^ (0.02)	–0.11^a^ (0.03)	–0.41^a^ (0.02)	–0.22^a^ (0.02)	–0.38^a^ (0.02)	–0.08^a^ (0.02)
Hispanic	–0.39^a^ (0.01)	–0.12^a^ (0.01)	–0.19^a^ (0.02)	–0.06^a^ (0.02)	–0.32^a^ (0.01)	–0.13^a^ (0.01)	–0.35^a^ (0.01)	–0.10^a^ (0.01)
**Population health controls**								
Diabetes prevalence	0.01^a^ (0)	0.01^a^ (0)	0 (0)	0.01^a^ (0)	0.01^a^ (0)	0.01^a^ (0)	0^c^ (0)	0^b^ (0)
Asthma prevalence	0.01^a^ (0)	–0.00 (0)	–0.01^a^ (0)	0 (0)	0.01^a^ (0)	–0.01^a^ (0)	0.01^a^ (0)	0 (0)
Coronary heart disease prevalence	–0.03^a^ (0)	–0.01^a^ (0)	–0.02^a^ (0)	–0.01^a^ (0)	–0.03^a^ (0)	–0.01^a^ (0)	–0.02^a^ (0)	0^c^ (0)
Checkup prevalence per 100	0.31^a^ (0.03)	–0.23^a^ (0.05)	0.17^a^ (0.05)	0.09 (0.09)	0.33^a^ (0.03)	–0.32^a^ (0.06)	0.23^a^ (0.03)	0.02 (0.06)
Binge drinking prevalence	–0.01^a^ (0.00)	0^a^ (0)	0^a^ (0)	–0.00 (0)	–0.01^a^ (0)	–0.01^a^ (0)	–0.01^a^ (0)	0^c^ (0)
**Economic control**								
Median household Income per $10 000	–0.02^a^ (0)	–0.01^a^ (0)	–0.01^a^ (0)	0^a^ (0)	–0.01^a^ (0)	0^a^ (0)	–0.01^a^ (0)	–0.01^a^ (0)
Observations	25 096	25 096	16 006	16 006	24 613	24 613	23 410	23 410
*R^2^*	0.15	0.51	0.06	0.32	0.13	0.44	0.15	0.45
State indicators^d^	No	Yes	No	Yes	No	Yes	No	Yes

^a^ *P* < .01.

^b^ *P* < .1.

^c^ *P* < .05.

^d^ Controls included across all specifications consist of population count, population density, Hispanic and Asian ethnicity, diabetes prevalence, asthma prevalence, coronary heart disease prevalence, checkup prevalence, binge drinking prevalence, and median household income. Controls include state indicators, which are binary indicators—1 for each state.

## Discussion

This study included 2 sets of descriptive evidence supportive of a health insurance redlining thesis. First, we found a significant inverse association between county-level non-Hispanic Black population and insurance carrier market participation. This finding suggests potential access barriers for non-Hispanic Black individuals because counties with larger non-Hispanic Black populations tended to have fewer participating insurance carriers. Lack of competition may present individuals with potentially fewer choices and with inflated premiums for plans on offer due to decreased competition.^17,18^ Given that most markets within our sample only had 1 to 3 insurance carriers, this lack of choice is likely to adversely affect beneficiaries rather than to helpfully provide external restrictions on an overabundance of choices.^23^

Second, our analyses found novel associations between census tract non-Hispanic Black population and the level of insurance network participation, such as the inclusion of physicians within that area. It is important to consider these findings in the context of prior work concerning racial and ethnic access gaps. As noted in the introduction, previous studies have identified racial access gaps in insurance coverage, primary care access, and access to freestanding emergency departments.^12,13,14,15,16^ Our findings indicated that insurer network inclusion or exclusion may present yet another compounding factor that would reduce access to clinics and physicians for non-Hispanic Black patients, and as a result increase overall racial and ethnic health care access disparities. These results would suggest an urgent need for the examination and enforcement of network adequacy standards within the ACA individual market exchanges.

In summary, we found statistically significant associations of regional non-Hispanic Black population prevalence with insurers market participation and network inclusion and exclusion decisions. While the effect sizes associated with these measures were reduced when controlling for additional factors pertaining to population health and household income, they still remained significant. These observations suggest that the actions by insurers within the individual ACA marketplaces may not only be perpetuating historical structural inequities that have resulted in well-documented health disparities across race and ethnicity, but that insurers may in fact be making strategic decisions as a direct function of a region’s racial makeup. Such market dynamics are concerning as they risk further growing existing racial inequities in health care access and in health outcomes, and they deserve further examination. Another aspect that future work may wish to explore is whether strategic decisions concerning market participation and insurance network inclusion and exclusion vary systematically across insurers. The examination of such behavioral heterogeneities appears particularly important to regulators who may wish to implement market-based solutions to the problem of limited selection, which disproportionately hurts racial and ethnic minority populations.

### Limitations

A number of study limitations are important to note. First, the present study was based on an observational study design that was only able to detect associations, not causation. Second, due to this design limitation, our results may be sensitive to residual confounding due to omitted variable bias. With that noted, we have sought to ameliorate the risk of such bias by including controls across population and demographic, population health, and economic factors. Our analyses have also sought to account for latent state policy and other differences that may have influenced insurers’ market participation and network inclusion decisions. This was done by means of including state indicators (or fixed effects) within our analyses. Third, our county-level insurer market analyses may not generalize to the states not included within our sample; and our census tract–level physician network breadth analyses may not generalize to specialties other than the 4 considered within our study, nor to less populous rural geographic areas, as our sample covered larger populous cities (eMethods in the Supplement). Fourth, it is not known how each of our outcomes relate to access to care or other adverse outcomes pertaining to cost and quality of care. Lastly, we note that the present study has used data from the inception year of the ACA individual exchanges, and it appears that further market examinations using more recent data, and potentially across other markets, could present important additions.

## Conclusions

Our findings suggest that strategic decisions by insurers may contribute toward markets with larger racial and ethnic minority populations, and specifically high percentages of non-Hispanic Black residents, having systematically fewer participating insurers and physicians with lower network inclusion. These findings call for further examination of potential insurance redlining within the ACA marketplaces.

## Notes

Supplement.
eMethods.
**eFigure 1.** Market Participation Sample **eFigure 2.** Provider Network Breadth Sample **eTable 1.** Number of County Markets per State **eTable 2.** Number of Census-tract Markets per State by Provider Specialty Sample **eTable 3.** Regression Model Estimates from County Level Sample Examining Association Between Number of Market Entrants and the Non-Hispanic Black Prevalence with the Inclusion of a State Level Medicaid Expansion Indicator **eTable 4.** Regression Model Estimates from County Level Sample Examining Association Between HHI and the Non-Hispanic Black Prevalence **eTable 5.** Quadratic Regression Model Estimates from County Level Sample Examining Association Between Number of Market Entrants and the Non-Hispanic Black Prevalence **eTable 6.** Pooled Regression Model Estimates from Provider Census Tract Sample
