# Supplementary material for: Association of County Race and Ethnicity Characteristics With Number of Insurance Carriers and Insurance Network Breadth
Source: JAMA Netw Open. 2022 Apr 27;5(4):e227404. doi: 10.1001/jamanetworkopen.2022.7404 (PMC9047637; doi:10.1001/jamanetworkopen.2022.7404)
Supplement: Supplement. — eMethods. eFigure 1. Market Participation Sample eFigure 2. Provider Network Breadth Sample eTable 1. Number of County Markets per State eTable 2. Number of Census-tract Markets per State by Provider Specialty Sample eTable 3. Regression Model Estimates from County Level Sample Examining Association Between Number of Market Entrants and the Non-Hispanic Black Prevalence with the Inclusion of a State Level Medicaid Expansion Indicator eTable 4. Regression Model Estimates from County Level Sample Examining Association Between HHI and the Non-Hispanic Black Prevalence eTable 5. Quadratic Regression Model Estimates from County Level Sample Examining Association Between Number of Market Entrants and the Non-Hispanic Black Prevalence eTable 6. Pooled Regression Model Estimates from Provider Census Tract Sample [file jamanetwopen-e227404-s001.pdf]

Supplemental Online Content

Linde S, Egede LE. Association of county race and ethnicity characteristics with number of insurance carriers and insurance network breadth. *JAMA Netw Open.* 2022;5(4):e227404. doi:10.1001/jamanetworkopen.2022.7404

eMethods.

eFigure 1. Market Participation Sample

eFigure 2. Provider Network Breadth Sample

eTable 1. Number of County Markets per State

eTable 2. Number of Census-tract Markets per State by Provider Specialty Sample

eTable 3. Regression Model Estimates from County Level Sample Examining Association Between Number of Market Entrants and the Non-Hispanic Black Prevalence with the Inclusion of a State Level Medicaid Expansion Indicator

eTable 4. Regression Model Estimates from County Level Sample Examining Association Between HHI and the Non-Hispanic Black Prevalence

eTable 5. Quadratic Regression Model Estimates from County Level Sample Examining Association Between Number of Market Entrants and the Non-Hispanic Black Prevalence

eTable 6. Pooled Regression Model Estimates from Provider Census Tract Sample

This supplemental material has been provided by the authors to give readers additional information about their work.

© 2022 Linde S et al. *JAMA Network Open.*

eMethods.

A. Additional Details on the Specification of Regression Models

The regression model specification for our first set of (county level) analyses is given by:

$$Number\ of\ Insurance\ Carriers_c = \alpha + \tau NHB_c + \boldsymbol{\beta X} + \boldsymbol{\phi_s} + \epsilon_c, \qquad (1)$$

where $Number\ of\ Insurance\ Carriers_c$ captures the number of health insurance carriers within county *c*, NHB_c is the non-Hispanic black prevalence in a given county, $\boldsymbol{X}$ denotes the other county level controls (previously described), and ϕ_s captures the state specific indicators.

The regression model for the second set of (census tract) analysis is given by:

$$Avg.Specialty\ Network\ Breadth_r = \alpha + \tau NHB_r + \boldsymbol{\beta X} + \boldsymbol{\phi_s} + \epsilon_r, \qquad (2)$$

where $Avg.Specialty\ Network\ Breadth_r$ is the average provider network breadth (for a given specialty) within census-track region *r.* All other variables in equation (2) are similarly defined to those in equation (1), but at the census tract (rather than county) level.

B. Additional Sensitivity Checks

Robustness to Use of HHI Outcome Instead of the Number of Health Insurance Carriers Count Used within Main County Market Participation Analysis

As an additional robustness check, we drew on carrier specific enrollment data from the individual exchanges in 2014 to construct Herfindahl-Hirschman Indices (HHI) for each county. The CMS issuer enrollment data that we used to construct this measure has a limitation in that it censors enrollment figures for plans with 10 or fewer enrolls within a given county. Given this limitation, we employ three different imputation approaches: (1)

© 2022 Linde S et al. *JAMA Network Open.*

impute censored enrollment values with “0”; (2) impute censored enrollment values with “5”; and (3) impute censored enrollment values with “10”. Once these enrollment figures were imputed we computed county specific market shares for each insurer and then used these to compute the HHI (which is theoretically bound between 1 = monopoly, and 0 = perfect competition).

Results from when we replaced our “Number of Health Insurance Carriers” count outcome with the HHI are provided within eTable 4. Looking at eTable 4 two things stand out. First, we note that the results are qualitatively similar when we use the HHI as our main outcome measure (instead of using the insurer count measure). That is, the results indicate that areas with higher non-Hispanic black population shares are significantly associated with less competitive, and more concentrated, insurance carrier markets ($p<0.01$ across all specifications). Second, we note similar results across all three sets of imputation approaches. This suggest that these findings are robust to alternative imputation strategies for when small enrollments are censored.

Robustness to Use of HHI Outcome Instead of the Number of Health Insurance Carriers Count Used within Main County Market Participation Analysis

As an additional robustness check, we drew on carrier specific enrollment data from the individual exchanges in 2014 to construct Herfindahl-Hirschman Indices (HHI) for each county. The CMS issuer enrollment data that we used to construct this measure has a limitation in that it censors enrollment figures for plans with 10 or fewer enrolls within a given county. Given this limitation, we employ three different imputation approaches: (1) impute censored enrollment values with “0”; (2) impute censored enrollment values with “5”; and (3) impute censored enrollment values with “10”. Once these enrollment figures

© 2022 Linde S et al. *JAMA Network Open.*

were imputed we computed county specific market shares for each insurer and then used these to compute the HHI (which is theoretically bound between 1 = monopoly, and 0 = perfect competition).

Results from when we replaced our “Number of Health Insurance Carriers” count outcome with the HHI are provided within eTable 4. Looking at eTable 4 two things stand out. First, we note that the results are qualitatively similar when we use the HHI as our main outcome measure (instead of using the insurer count measure). That is, the results indicate that areas with higher non-Hispanic black population shares are significantly associated with less competitive, and more concentrated, insurance carrier markets ($p<0.01$ across all specifications). Second, we note similar results across all three sets of imputation approaches. This suggest that these findings are robust to alternative imputation strategies for when small enrollments are censored.

Robustness to An Alternative Quadratic Functional Form Specification

Figure 1 within the main text suggests that the association between markets having one health insurance carriers and two, or three, health insurance carries within a given market is visibly associated with the non-Hispanic black prevalence of the county in question. However, the effect of this seems less pronounced for markets with larger number of competitors. This suggest that an alternative (quadratic) functional form relating the number of health insurance carriers and the non-Hispanic black prevalence of a market might provide a better fit. eTable 5 indicates qualitatively similar results to our main results. Additionally, while the quadratic terms are broadly statistically significant, the model fit is improved only marginally, if at all. As such, we report the results from the simpler (linear) functional form specification within our main results of

© 2022 Linde S et al. *JAMA Network Open.*

the paper.

Robustness to Pooled Specifications of Census Tract Provider Network Breadth Analysis

Rather than present results as stratified by specialty, eTable 6 presents pooled results across specialty. In columns (1) and (2) we do not allow for any heterogeneity across specialties, however, in columns (3) and (4) this heterogeneity is allowed by the inclusion of specialty interactions (where pediatrics represents the omitted specialty category) with the non-Hispanic black prevalence measure. Columns (3) and (4) indicate significant heterogeneities across specialties, and results are qualitatively similar to our main (specialty stratified) results within the text.

© 2022 Linde S et al. *JAMA Network Open.*

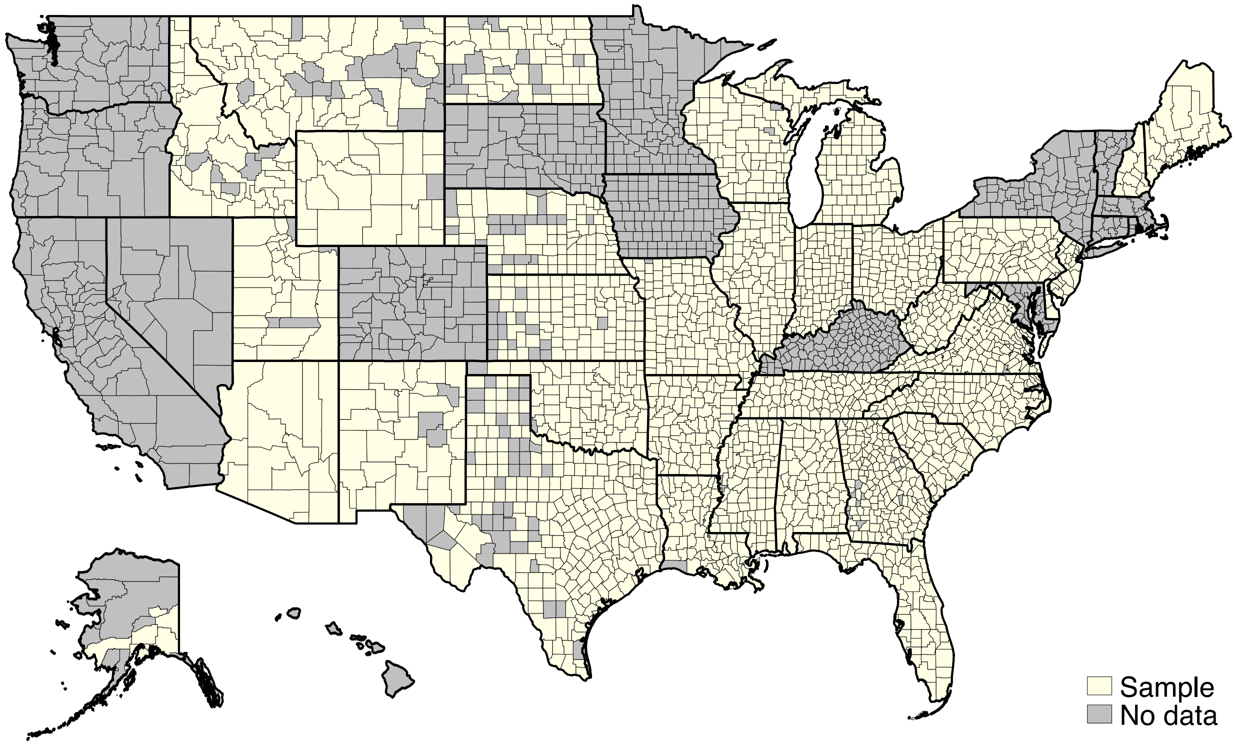

eFigure 1. Market Participation Sample

Market participation sample consists of counties marked in yellow. These markets cover 34 states.

© 2022 Linde S et al. *JAMA Network Open.*

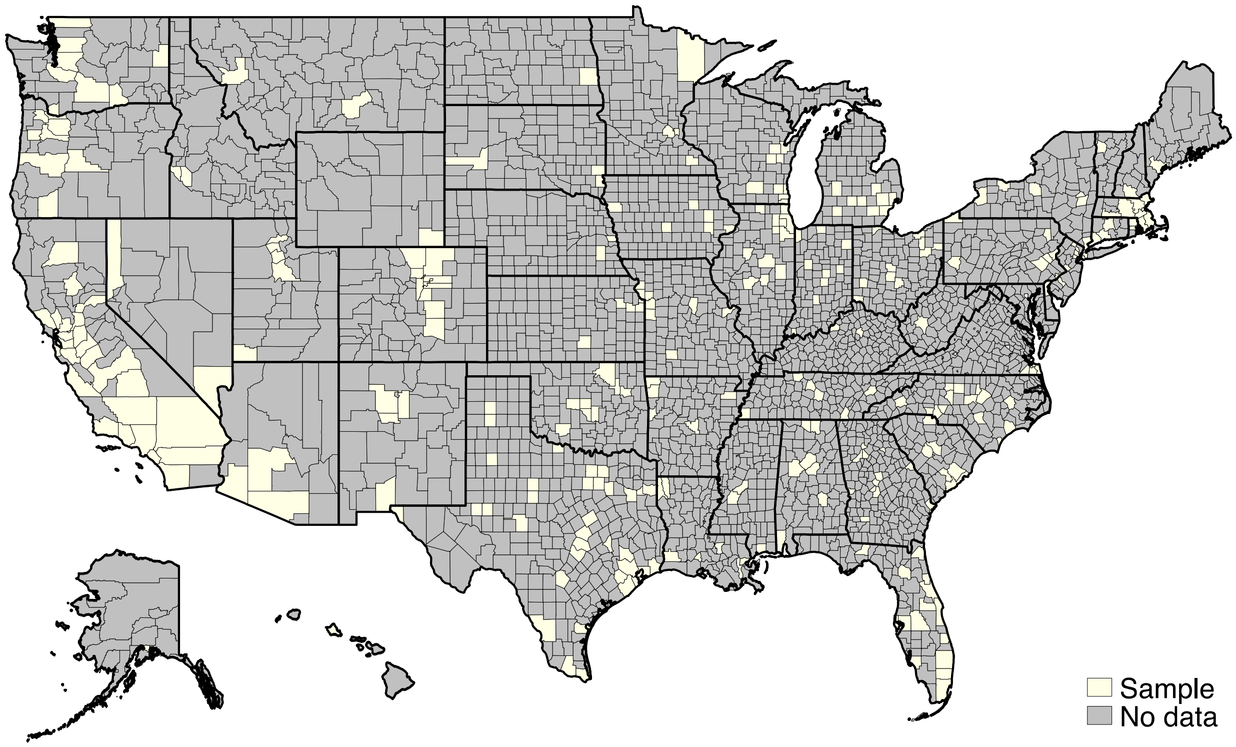

eFigure 2. Provider Network Breadth Sample

Provider network breadth sample consists of census-tract regions contained within the counties marked in yellow. These markets cover all 50 states and Washington DC, and they are based on the 500 largest US cities contained within the CDC PLACES database.

© 2022 Linde S et al. *JAMA Network Open.*

eTable 1. Number of County Markets per State

	N	Percent	Cum.
AK	11	0.48	0.48
AL	67	2.95	3.44
AR	75	3.30	6.74
AZ	15	0.66	7.40
DE	3	0.13	7.53
FL	67	2.95	10.48
GA	149	6.56	17.05
ID	39	1.72	18.77
IL	102	4.49	23.26
IN	92	4.05	27.31
KS	90	3.96	31.28
LA	63	2.78	34.05
ME	16	0.70	34.76
MI	82	3.61	38.37
MO	114	5.02	43.39
MS	81	3.57	46.96
MT	40	1.76	48.72
NC	100	4.41	53.13
ND	41	1.81	54.93
NE	73	3.22	58.15
NH	10	0.44	58.59
NJ	21	0.93	59.52
NM	30	1.32	60.84
OH	88	3.88	64.71
OK	75	3.30	68.02
PA	67	2.95	70.97
SC	46	2.03	73.00
TN	95	4.19	77.18
TX	216	9.52	86.70
UT	25	1.10	87.80
VA	130	5.73	93.52
WI	70	3.08	96.61
WV	55	2.42	99.03
WY	22	0.97	100.00

© 2022 Linde S et al. *JAMA Network Open.*

Total	2270	100.00	

© 2022 Linde S et al. *JAMA Network Open.*

eTable 2. Number of Census-tract Markets per State by Provider Specialty Sample

Sample:	**Family Medicine**			**General Practice**			**Internist**		
State	N	Percent	Cum.	N	Percent	Cum.	N	Percent	Cum.
AK	49	0.20	0.20	37	0.23	0.23	37	0.15	0.15
AL	307	1.22	1.42	208	1.30	1.53	287	1.17	1.32
AR	115	0.46	1.88	95	0.59	2.12	105	0.43	1.74
AZ	974	3.88	5.76	816	5.10	7.22	968	3.93	5.68
CA	4905	19.54	25.30	4122	25.75	32.98	4881	19.83	25.51
CO	652	2.60	27.90	270	1.69	34.66	629	2.56	28.06
CT	202	0.80	28.71	113	0.71	35.37	211	0.86	28.92
DC	175	0.70	29.40	117	0.73	36.10	175	0.71	29.63
DE	24	0.10	29.50	8	0.05	36.15	24	0.10	29.73
FL	1231	4.91	34.40	980	6.12	42.27	1214	4.93	34.66
GA	411	1.64	36.04	213	1.33	43.60	394	1.60	36.26
HI	216	0.86	36.90	192	1.20	44.80	217	0.88	37.14
IA	189	0.75	37.66	56	0.35	45.15	164	0.67	37.81
ID	63	0.25	37.91	46	0.29	45.44	60	0.24	38.05
IL	1196	4.77	42.67	832	5.20	50.64	1194	4.85	42.90
IN	484	1.93	44.60	216	1.35	51.99	471	1.91	44.82
KS	290	1.16	45.76	89	0.56	52.54	269	1.09	45.91
KY	254	1.01	46.77	187	1.17	53.71	250	1.02	46.93
LA	333	1.33	48.10	166	1.04	54.75	318	1.29	48.22
MA	461	1.84	49.93	182	1.14	55.89	465	1.89	50.11
MD	181	0.72	50.65	172	1.07	56.96	195	0.79	50.90
ME	20	0.08	50.73	1	0.01	56.97	20	0.08	50.98
MI	668	2.66	53.39	310	1.94	58.90	669	2.72	53.70
MN	309	1.23	54.63	128	0.80	59.70	295	1.20	54.90
MO	394	1.57	56.20	163	1.02	60.72	375	1.52	56.42
MS	64	0.26	56.45	6	0.04	60.76	63	0.26	56.68
MT	43	0.17	56.62	16	0.10	60.86	43	0.17	56.85
NC	747	2.98	59.60	433	2.71	63.56	724	2.94	59.79
ND	25	0.10	59.70	25	0.16	63.72	25	0.10	59.90
NE	203	0.81	60.51	82	0.51	64.23	189	0.77	60.66
NH	38	0.15	60.66	15	0.09	64.33	44	0.18	60.84
NJ	284	1.13	61.79	90	0.56	64.89	297	1.21	62.05
NM	205	0.82	62.61	172	1.07	65.96	196	0.80	62.84
NV	275	1.10	63.70	310	1.94	67.90	332	1.35	64.19
NY	2406	9.59	73.29	1357	8.48	76.38	2429	9.87	74.06
OH	756	3.01	76.30	407	2.54	78.92	754	3.06	77.13
OK	427	1.70	78.00	193	1.21	80.13	395	1.60	78.73
OR	324	1.29	79.30	273	1.71	81.83	326	1.32	80.06
PA	247	0.98	80.28	103	0.64	82.48	244	0.99	81.05
RI	83	0.33	80.61	57	0.36	82.83	97	0.39	81.44
SC	153	0.61	81.22	131	0.82	83.65	157	0.64	82.08
SD	55	0.22	81.44	22	0.14	83.79	52	0.21	82.29
TN	480	1.91	83.35	365	2.28	86.07	480	1.95	84.24
TX	2558	10.19	93.54	1164	7.27	93.34	2341	9.51	93.75
UT	226	0.90	94.45	156	0.97	94.31	183	0.74	94.49
VA	483	1.92	96.37	369	2.31	96.62	465	1.89	96.38
VT	10	0.04	96.41	10	0.06	96.68	11	0.04	96.43

© 2022 Linde S et al. *JAMA Network Open.*

WA	483	1.92	98.33	309	1.93	98.61	462	1.88	98.31
WI	380	1.51	99.85	194	1.21	99.83	382	1.55	99.86
WV	22	0.09	99.94	15	0.09	99.92	22	0.09	99.95
WY	16	0.06	100.0	13	0.08	100.0	13	0.05	100.0
Total	25,096	100.0		16,006	100.0		24,613	100.0	

© 2022 Linde S et al. *JAMA Network Open.*

eTable 3. Regression Model Estimates from County Level Sample Examining Association Between Number of Market Entrants and the Non-Hispanic Black Prevalence with the Inclusion of a State Level Medicaid Expansion Indicator.

	(1)	(2)	(3)
	Number of Health Insurance Carriers	Number of Health Insurance Carriers	Number of Health Insurance Carriers
Non-Hispanic Black (%)	-1.86[a]	-0.54[a]	-0.48[a]
	(0.13)	(0.18)	(0.18)
Medicaid Expansion Indicator	0.74[a]<br>(0.05)	0.69[a]<br>(0.05)	0.68[a]<br>(0.05)
Controls Included	X	X	X
Observations	2,270	2,270	2,270
R-squared	0.26	0.34	0.34

Note: Robust standard errors are reported within the in parentheses; statistical significance is denoted as: [a] p<0.01, [b] p<0.05, [c] p<0.1. Controls included across all specifications consist of: Population Count, Age 18 and Younger (%), Age 65 and Older (%), Hispanic (%), Asian (%), Rural (%), Premature Death, Diabetes Prevalence, Preventable Hospital Stays, and Median Household Income.

© 2022 Linde S et al. *JAMA Network Open.*

eTable 4. Regression Model Estimates from County Level Sample Examining Association Between HHI and the Non-Hispanic Black Prevalence.

	Censored Enrollments Imputed with 0			Censored Enrollments Imputed with 5			Censored Enrollments Imputed with 10		
	(1)	(2)	(3)	(4)	(5)	(6)	(7)	(8)	(9)
	HHI	HHI	HHI	HHI	HHI	HHI	HHI	HHI	HHI
Non-Hispanic Black (%)	0.12[a] (0.03)	0.16[a] (0.04)	0.14[a] (0.04)	0.13[a] (0.03)	0.15[a] (0.03)	0.12[a] (0.03)	0.12[a] (0.03)	0.13[a] (0.03)	0.10[a] (0.03)
Controls Included	X	X	X	X	X	X	X	X	X
Observations	2,270	2,270	2,270	2,270	2,270	2,270	2,270	2,270	2,270
R-squared	0.56	0.58	0.58	0.57	0.58	0.59	0.56	0.57	0.58
State Indicators	X	X	X	X	X	X	X	X	X

Note: Robust standard errors are reported within the in parentheses; statistical significance is denoted as: [a] $p<0.01$, [b] $p<0.05$, [c] $p<0.1$. Controls are included in a sequential fashion as within Table 2 (columns 4-6) of the main text; and the same controls are used here as within Table 2 of the main text.

© 2022 Linde S et al. *JAMA Network Open.*

eTable 5. Quadratic Regression Model Estimates from County Level Sample Examining Association Between Number of Market Entrants and the Non-Hispanic Black Prevalence.

	(1)	(2)	(3)	(4)	(5)	(6)
	Number of Health Insurance Carriers	Number of Health Insurance Carriers	Number of Health Insurance Carriers	Number of Health Insurance Carriers	Number of Health Insurance Carriers	Number of Health Insurance Carriers
Non-Hispanic Black (%)	-3.77[a]	-2.09[a]	-2.05[a]	-1.13[a]	-1.21[a]	-1.02[b]
	(0.38)	(0.39)	(0.39)	(0.42)	(0.42)	(0.42)
Non-Hispanic Black^2	3.02[a]	2.39[a]	2.51[a]	0.87[c]	1.14[b]	1.10[b]
	(0.61)	(0.58)	(0.57)	(0.52)	(0.51)	(0.51)
Controls Included	X	X	X	X	X	X
Observations	2,270	2,270	2,270	2,270	2,270	2,270
R-squared	0.20	0.28	0.28	0.59	0.61	0.61
State Indicators	-	-	-	X	X	X

Note: Robust standard errors are reported within the in parentheses; statistical significance is denoted as: [a] $p<0.01$, [b] $p<0.05$, [c] $p<0.1$. Controls are included in a sequential fashion as within Table 2 of the main text; and the same controls are used here as within Table 2 of the main text.

© 2022 Linde S et al. *JAMA Network Open.*

eTable 6. Pooled Regression Model Estimates from Provider Census Tract Sample.

	(1)	(2)	(3)	(4)
	Provider Network Breadth (%)	Provider Network Breadth (%)	Provider Network Breadth (%)	Provider Network Breadth (%)
Non-Hispanic Black (%)	-0.26[a]	-0.09[a]	-0.26[a]	-0.09[a]
	(0.01)	(0.01)	(0.01)	(0.01)
Family Medicine [c] Non-Hispanic Black			0.00<br>(0.01)	0.00<br>(0.01)
General Practice [c] Non-Hispanic Black			-0.06[a]<br>(0.01)	-0.06[a]<br>(0.01)
Internist [c] Non-Hispanic Black			0.02[a]<br>(0.01)	0.02[a]<br>(0.01)
Controls Included	X	X	X	X
Specialty Indicators	-	-	X	X
Observations	89,125	89,125	89,125	89,125
R-squared	0.12	0.38	0.16	0.42
State Indicators	-	X	-	X

Note: Robust standard errors are reported within the in parentheses; statistical significance is denoted as: [a] p<0.01, [b] p<0.05, [c] p<0.1. Controls included across all specifications consist of: Population Count, Population Density, Hispanic (%), Asian (%), Diabetes Prevalence, Asthma Prevalence, Coronary Heart Disease Prevalence, Checkup Prevalence, Binge Drinking Prevalence, and Median Household Income. The omitted specialty category is pediatrics within the results for columns 3 and 4. Columns 2 and 4 also control for state indicators.

© 2022 Linde S et al. *JAMA Network Open.*
